# Magnesium inhibits peritoneal calcification as a late-stage characteristic of encapsulating peritoneal sclerosis

**DOI:** 10.1038/s41598-023-43657-y

**Published:** 2023-09-28

**Authors:** Seishi Aihara, Shunsuke Yamada, Shumei Matsueda, Akinori Nagashima, Kumiko Torisu, Takanari Kitazono, Toshiaki Nakano

**Affiliations:** 1 Department of Medicine and Clinical Science, Graduate School of Medical Sciences, Kyushu University, 3-1-1 Maidashi, Higashi-ku, Fukuoka, 8128582 Japan; 2 Department of Nephrology, Karatsu Red Cross Hospital, Saga, Japan

**Keywords:** Kidney diseases, Renal replacement therapy

## Abstract

Peritoneal calcification is a prominent feature of the later stage of encapsulating peritoneal sclerosis (EPS) in patients undergoing long-term peritoneal dialysis (PD). However, the pathogenesis and preventive strategy for peritoneal calcification remain unclear. Peritoneum samples from EPS patients were examined histologically. Peritoneal calcification was induced in mice by feeding with an adenine-containing diet combined with intraperitoneal administration of lipopolysaccharide and a calcifying solution containing high calcium and phosphate. Excised mouse peritoneum, human mesothelial cells (MeT5A), and mouse embryonic fibroblasts (MEFs) were cultured in calcifying medium. Immunohistochemistry confirmed the appearance of osteoblastic differentiation-marker-positive cells in the visceral peritoneum from EPS patients. Intraperitoneal administration of magnesium suppressed peritoneal fibrosis and calcification in mice. Calcifying medium increased the calcification of cultured mouse peritoneum, which was prevented by magnesium. Calcification of the extracellular matrix was accelerated in Met5A cells and MEFs treated with calcification medium. Calcifying medium also upregulated osteoblastic differentiation markers in MeT5A cells and induced apoptosis in MEFs. Conversely, magnesium supplementation mitigated extracellular matrix calcification and phenotypic transdifferentiation and apoptosis caused by calcifying conditions in cultured MeT5A cells and MEFs. Phosphate loading contributes to the progression of EPS through peritoneal calcification and fibrosis, which can be prevented by magnesium supplementation.

## Introduction

Encapsulating peritoneal sclerosis (EPS), characterized by peritoneal fibrosis, angiogenesis, and fibrin accumulation, is a lethal complication in patients undergoing long-term peritoneal dialysis (PD)^1^. In the later stage of EPS, the surface of the peritoneum becomes calcified^2, 3^, typically involving hydroxyapatite deposition on the superficial layer of the peritoneum; however, the underlying pathologies remain unknown.

Ectopic calcification, especially vascular calcification (VC), is highly prevalent and associated with increased morbidity and mortality in patients with chronic kidney disease (CKD)^4, 5^. VC is actively regulated and associated with cell-mediated processes, including the phenotypic conversion of vascular smooth muscle cells (VSMCs) into osteoblast-like cells, the apoptosis and necrosis of VSMCs, and the formation of calciprotein particles (CPPs), a recently identified culprit of VC mainly composed of calcium (Ca), phosphate, and fetuin-A^6^. Hyperphosphatemia and phosphate overload are the most potent stimulators of ectopic calcification and VC, while hypercalcemia also contributes to the progression of VC^7^. Transperitoneal phosphate diffusion into the peritoneal dialysate is critical for phosphate elimination in patients receiving PD, inevitably exposing mesothelial cells lining the peritoneal cavity and sub-mesothelial fibroblasts to constant phosphate flow. Mesothelial cells and fibroblasts often undergo phenotypic switching in response to environmental changes and may thus play significant roles in the pathogenesis of peritoneal calcification in response to high phosphate and Ca conditions. Notably, CPPs are present in the PD fluid^8^. These results suggest that the peritoneum is constantly at risk of ectopic calcification, potentially leading to peritoneal calcification in PD patients.

Recent studies revealed that magnesium (Mg) inhibited phosphate-induced VC in vivo and in vitro^9^. Given that peritoneal calcification in PD patients is caused by persistent peritoneal exposure to a high-Ca, high-phosphate environment, Mg may be able to prevent peritoneal calcification in the later stage of EPS. Mg has also been shown to retard inflammation-driven renal and lung fibrosis^10, 11^. Considering the underlying pathology of EPS, intraperitoneal Mg supplementation may be an effective treatment option for patients at heightened risk of EPS.

The present study had two aims. First, we determined if inorganic phosphate loading accelerated peritoneal calcification, as a prominent feature of later-stage EPS, in mouse and human tissues and cultured peritoneum-constituting cells. Second, we determined if Mg supplementation could prevent the progression of peritoneal fibrosis and calcification in in vivo, ex vivo, and in vitro experiments.

## Results

### Osteoblastic differentiation in the peritoneum in patients with EPS

**Figure 1:**
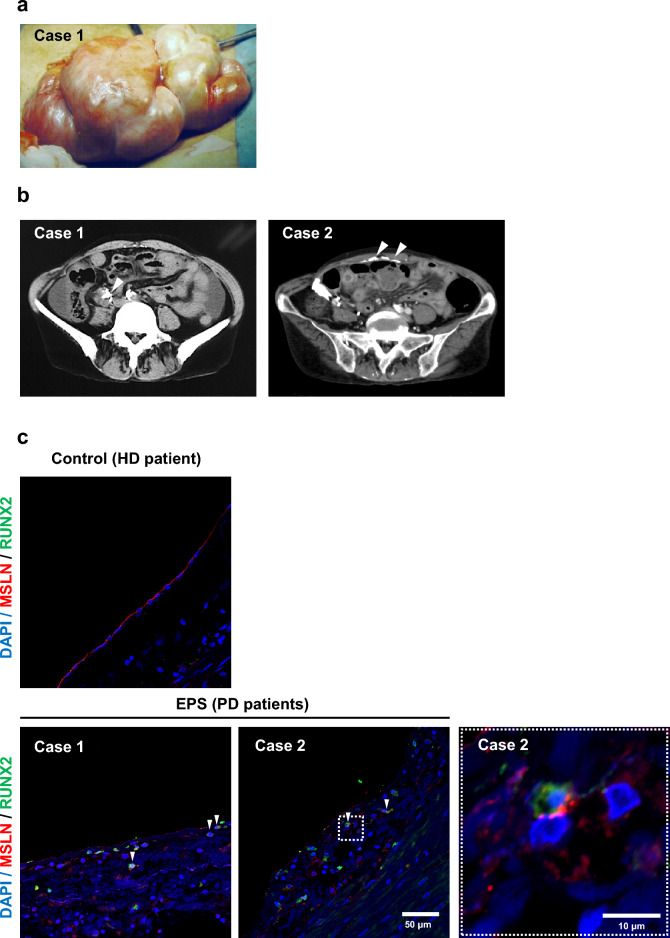
Encapsulating peritoneum in patients and osteoblastic differentiation-marker-positive cells in peritoneum in patients with encapsulating peritoneal sclerosis and peritoneal calcification. (**a**) Macroscopic image of encapsulating peritoneum in a patient with encapsulating peritoneal sclerosis (EPS) (case 1). (**b**) Computed tomography images of abdomen in patients with EPS (cases 1 and 2). White arrows indicate calcification of visceral and parietal peritoneum. (**c**) Representative photomicrographs of dual immunohistochemical staining of mesothelin (MSLN) and runt-related transcription factor 2 (RUNX2) in the peritoneum in two peritoneal dialysis (PD) patients with EPS and one hemodialysis (HD) patient (control). Red, anti-MSLN antibody; green, anti-RUNX2 antibody; blue, DAPI. RUNX2 and MSLN co-localization indicated by arrowheads. White square indicates cropped image of MSLN and RUNX2 double-positive cells. Double-positive cells were found in PD patients but not in HD patients. Scale bars 50 µm in original images and 10 µm in cropped image.

Encapsulating peritoneum and abdominal computed tomography images indicating peritoneal calcification are shown in Fig. 1a,b. We determined if peritoneum samples from PD patients diagnosed with EPS with peritoneal calcification expressed osteoblastic differentiation markers. We initially identified the appearance of runt-related transcription factor 2 (RUNX2)-positive cells in the peritoneal interstitium and then investigated their origin. Immunohistochemistry showed that mesothelin (MSLN) expression was reduced in the EPS samples compared with the control sample, and MSLN and RUNX2 were co-localized in the visceral peritoneum in the EPS cases (Fig. 1c). These results suggest that RUNX2-positive cells may originate from mesothelial cells. However, no obvious α-smooth muscle actin (α-SMA) and RUNX2 double-positive cells were observed in the peritoneum in the EPS cases (Supplementary Fig. S1). MSLN-positive cells mimicked the morphology of macrophages (Fig. 1c) and we therefore also conducted double immunofluorescence studies. RAW 246.7 cultured mouse macrophages were stained with anti-F4/80 antibody (Supplementary Fig. S1). However, no double-positive cells, stained with both anti-F/80 antibody and anti-MSLN antibody, were found in a peritoneum specimen derived from an EPS patient (Supplementary Fig. S1).

### Effects of calcifying solution and Mg supplementation on Ca content in a mouse model of peritoneal calcification with acute peritonitis and CKD

**Figure 2:**
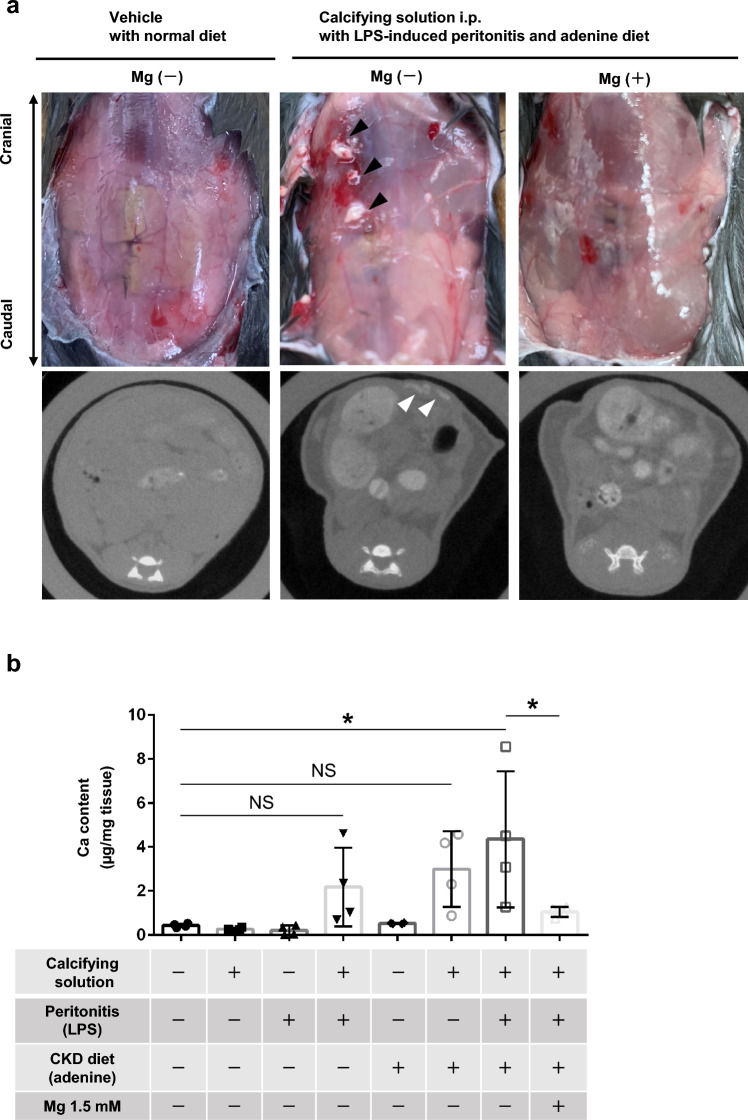

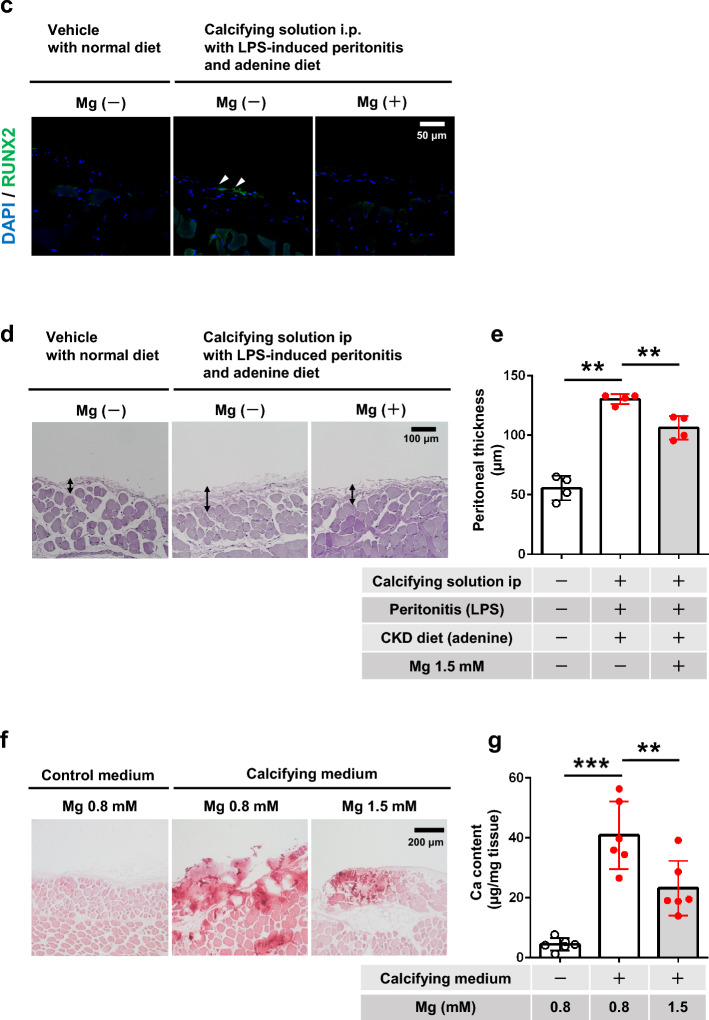
Impacts of calcifying conditions and magnesium on calcification, fibrosis, and phenotypic change in mouse peritoneum induced by lipopolysaccharide and chronic kidney disease (in vivo and ex vivo). (**a**) Representative photographs of calcified parietal peritoneum and computed tomography images. Black and white arrowheads indicate spotty calcification. (**b**) Quantification of calcium content in the parietal peritoneum (n = 4 per group). (**c**) Representative confocal immunofluorescence microscopic images of runt-related transcription factor 2 (RUNX2) in mouse parietal peritoneum. Green, anti-RUNX2 antibody; blue, DAPI. White arrows indicate RUNX2-positive cells in the peritoneum. Scale bars 50 µm. (**d**) Representative images of periodic acid-Schiff staining of mouse parietal peritoneum. Black double arrows indicate thickness of parietal peritoneum. Scale bars 100 µm. (**e**) Comparison of average thickness of peritoneal fibrosis among groups (n = 4 per group). (**f**) Representative histological image of mouse parietal peritoneum stained by Alizarin Red (ex vivo). Scale bars 200 μm. (**g**) Quantitative evaluation of calcium content in peritoneum in ex vivo experiment (n = 6 per group). *P < 0.05, **P < 0.01, ***P < 0.001, *NS* not significant, *CKD* chronic kidney disease.

We next assessed if injection of Ca- and phosphate-rich calcifying solution could induce peritoneal calcification in mice. Analyses of Ca content showed that intraperitoneal injection of calcifying solution alone did not induce peritoneal calcification; however, peritoneal Ca content tended to increase when administered with intraperitoneal lipopolysaccharide (LPS) or an adenine diet. Moreover, peritoneal calcification was considerably enhanced when phosphate-rich calcifying solution was administered in combination with LPS administration and an adenine diet. The Ca content in the peritoneum increased significantly, as confirmed by computed tomography (Fig. 2a,b). Notably, Mg supplementation decreased peritoneal calcification (Fig. 2a,b). Immunostaining showed the appearance of RUNX2-positive cells in the calcified peritoneum and their absence in the Mg-treated group (Fig. 2c). Peritoneal thickness was significantly increased in the calcified peritoneum group but was suppressed by Mg treatment (Fig. 2d,e). A CKD-inducing diet caused body weight loss, which tended to be inhibited by Mg administration (Supplementary Fig. S2). Intraperitoneal Mg supplementation did not affect serum Mg concentrations (Supplementary Fig. S2).

We carried out serum biochemistry analyses to confirm if the adenine diet caused CKD (Supplementary Fig. S2). Renal interstitial fibrosis was also confirmed histologically by Sirius Red staining. Serum levels of creatinine and urea nitrogen were not increased in the adenine-diet groups (Supplementary Fig. S2). However, serum levels of cystatin C, as another marker of kidney functions unaffected by skeletal muscle volume, were significantly increased in the adenine-diet groups (Supplementary Fig. S2). Hyperphosphatemia was also induced by feeding an adenine diet (Supplementary Fig. S2), while Ca levels tended to decrease in the adenine-diet groups (Supplementary Fig. S2). Sirius Red staining of the kidneys showed severe and diffuse tubular atrophy and interstitial fibrosis in the adenine-diet-induced CKD group (Supplementary Fig. S2). These results suggest that CKD was induced by an adenine diet.

### Effects of calcifying solution and Mg supplementation on peritoneal calcification

In the above in vivo experiment, the peritoneum was not continuously exposed to calcifying solution because the solution administered intraperitoneally was absorbed. We therefore investigated if persistent exposure to calcifying medium promoted peritoneal calcification using an ex vivo model. Incubation in calcifying medium for 72 h induced significant peritoneal calcification, as evidenced by a significantly increased Ca content in the peritoneum (Fig. 2f). Conversely, peritoneal calcification was reduced by calcifying medium with a high Mg concentration (1.5 mM) (Fig. 2g).

### Impact of calcifying medium and Mg on osteoblastic changes and cell death in cultured mesothelial cells

**Figure 3:**
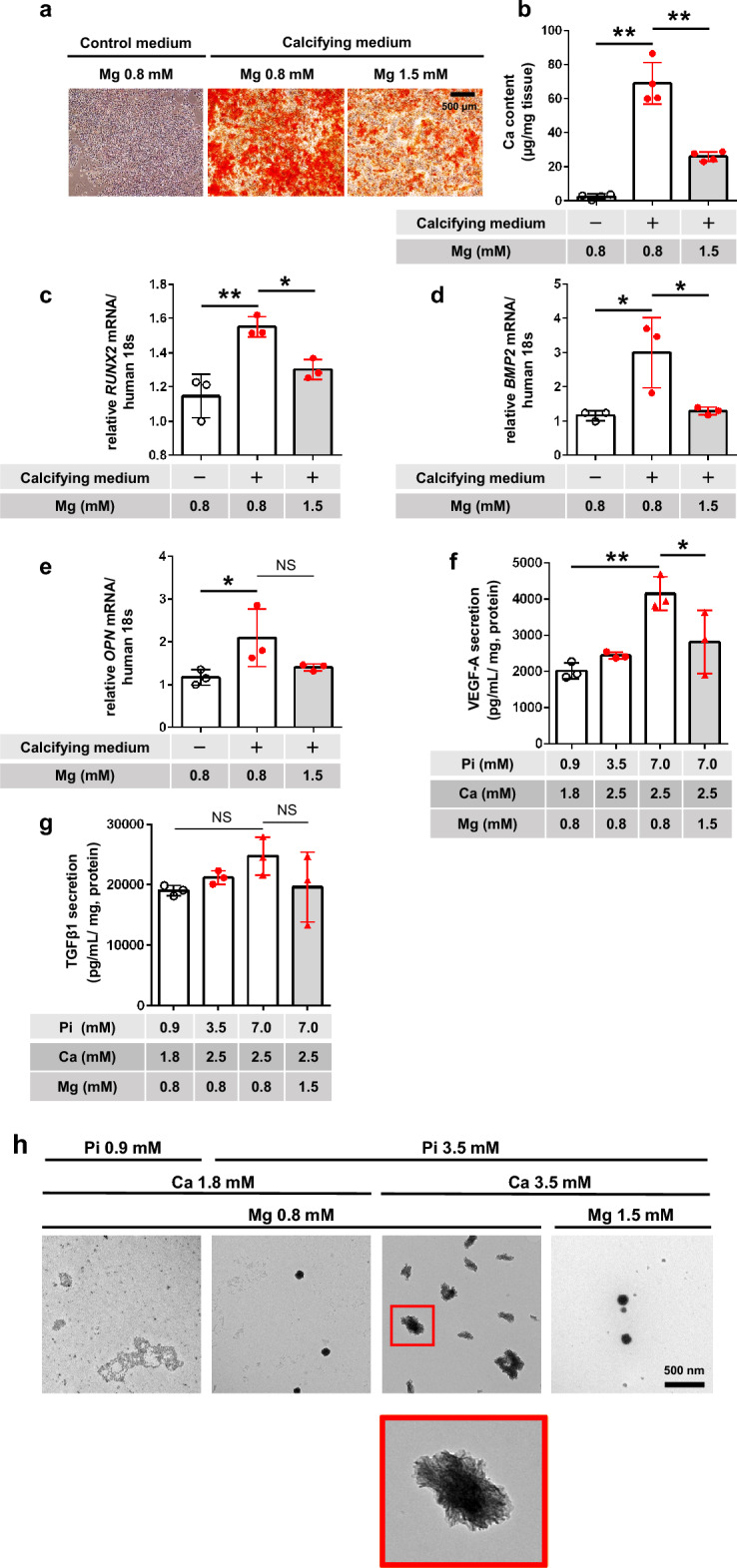
Impact of calcifying medium and magnesium on calcification and phenotypic change in cultured mesothelial cells (in vitro). (**a**) Representative photomicrographs of Alizarin red-stained cultured MeT5A cells. Scale bars 500 μm. (**b**) Quantitative analysis of calcification in MeT5A cells (n = 4 per group). Relative mRNA expression levels of (**c**) runt-related transcription factor 2 (*RUNX2*), (**d**) bone morphogenetic protein 2 (*BMP2*), and (**e**) osteopontin (*OPN*) determined by real-time polymerase chain reaction in MeT5A cells (n = 3 for each). Secretion of (**f**) vascular endothelial growth factor-A (VEGF-A) and (**g**) transforming growth factor-β1 (TGFβ1) corrected for protein (mg) in MeT5A cells incubated with calcifying medium, measured by enzyme-linked immunosorbent assay. *P < 0.05, **P < 0.01, *NS* not significant. (**h**) Representative images of mature calciprotein particles captured by transmission electron microscopy. Scale bars 500 nm. Red square indicates magnified image of mature calciprotein particle.

We investigated the role of peritoneal mesothelial cells in the pathogenesis of peritoneal calcification using MeT5A cells. The Ca content was significantly increased by calcifying medium and this was reduced by a high Mg-containing solution (Fig. 3a,b). Moreover, calcifying medium increased mRNA levels of the osteoblastic differentiation markers *RUNX2*, bone morphogenetic protein 2 (*BMP2*), and osteopontin (*OPN*) in MeT5A cells (Fig. 3c–e). The increased *RUNX2* and *BMP2* mRNA levels were suppressed by high Mg (Fig. 3c,d), while increased *OPN* mRNA levels tended to be suppressed by high Mg (Fig. 3e). We then determined if the calcifying environment-stimulated mesothelial cells affected other EPS pathologies, angiogenesis, and fibrosis, via paracrine actions. Analysis of Met5A cell supernatant showed that high-phosphate calcifying medium promoted vascular endothelial growth factor-A (VEGF-A) secretion, associated with angiogenesis, which was suppressed by high Mg conditions (Fig. 3f). Calcifying medium also tended to increase the secretion of transforming growth factor-β1 (TGFβ1), involved in fibrosis, which tended to be reversed by 1.5 mM Mg (Fig. 3g). Trypan blue staining indicated that calcifying medium decreased Met5A cell viability, which was canceled by calcifying medium containing 1.5 mM Mg (Supplementary Fig. S3). MeT5A cells were incubated with normal Ca concentration medium with high phosphate (1.8 mM Ca, 3.5 mM phosphate) or calcifying medium (2.5 mM Ca, 3.5 mM phosphate) for 72 h. Calcifying medium only induced calcification in MeT5A cells in the presence of high Ca concentration (Supplementary Fig. S3). The above animal study showed that peritoneal calcification was only promoted when mice were treated with LPS (Fig. 2b). We further investigated the effect of inflammation on mesothelial injury by stimulating MeT5A cells with conditioned medium from 24 h LPS-activated RAW 264.7 cells. LPS-activated macrophage supernatant significantly decreased the viability of MeT5A cells (Supplementary Fig. S3).

We also confirmed the presence and formation of CPPs in the culture medium using transmission electron microscopy. Calcifying medium promoted the formation of mature CPPs, but no mature CPPs were detected in calcifying medium containing 1.5 mM Mg (Fig. 3h).

### Impact of calcifying medium and Mg on transdifferentiation and apoptosis of cultured fibroblasts

**Figure 4:**
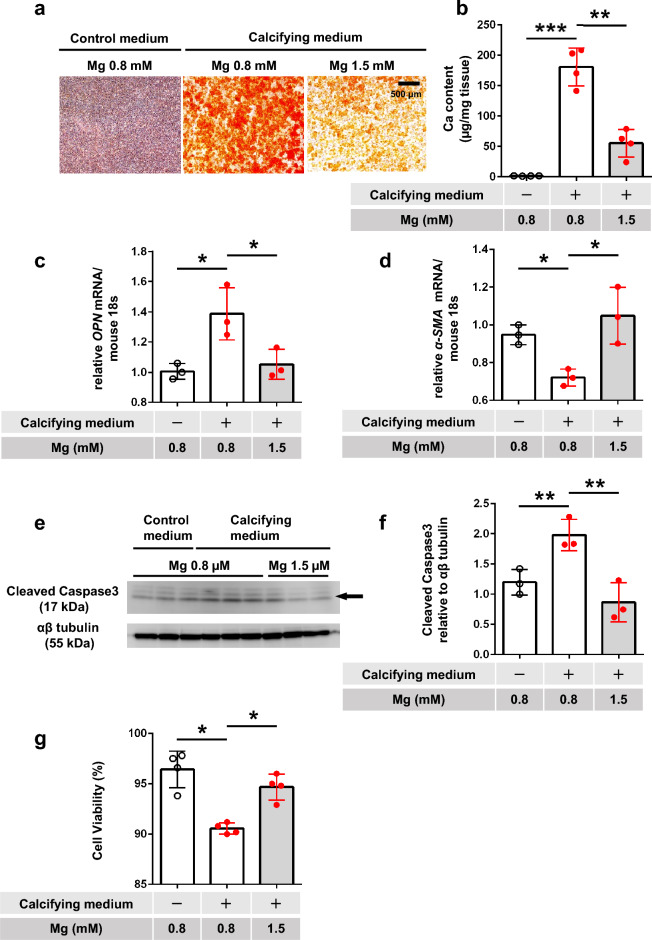
Impact of calcifying medium and magnesium on calcification and phenotypic change and apoptosis in cultured fibroblasts (in vitro). (**a**) Representative photographs of Alizarin red-stained cultured mouse embryonic fibroblasts (MEFs). Scale bars 500 μm. (**b**) Quantitative analysis of calcification in MEFs (n = 4 per group). Relative mRNA expression levels of (**c**) osteopontin (OPN) and (**d**) α-smooth muscle actin (SMA) in MEFs determined by real-time polymerase chain reaction (n = 3 per group). (**e**) Western blotting of cleaved caspase 3 protein in MEFs. Upper panel, cleaved caspase 3; lower panel, αβ tubulin (internal control). (**f**) Quantification of cleaved caspase 3 protein normalized to αβ tubulin (n = 3 each). (**g**) Cell viability determined by trypan blue staining (n = 4). *P < 0.05, **P < 0.01, ***P < 0.001, *NS* not significant.

We then investigated the role of fibroblasts in peritoneal calcification. Calcifying medium significantly increased calcification in MEFs compared with the control medium, and this calcification was suppressed by a high-Mg medium (Fig. 4a,b). The calcifying medium had no significant effects on *RUNX2* and *BMP2* mRNA levels (data not shown), but increased *OPN* mRNA levels, and this increase was canceled by high Mg (Fig. 4c). Meanwhile, α-SMA mRNA levels were significantly reduced by the calcifying medium, but maintained by calcifying medium containing 1.5 mM Mg (Fig. 4d), suggesting that Mg inhibited the transdifferentiation of MEFs and maintained their original phenotype. Expression of cleaved caspase 3, determined by western blotting, was enhanced by calcifying medium, and this effect was not prevented by medium containing 1.5 mM Mg (Fig. 4e,f, original blots/gels presented in Supplementary Fig. S4). Trypan blue staining showed that the calcifying medium significantly decreased cell viability, and this was reversed by calcifying medium containing 1.5 mM Mg (Fig. 4g).

### Impact of calcifying medium on calcification of cultured macrophages

Finally, we investigated the effect of calcifying medium and Mg on the calcification of cultured macrophages. Phenotypic changes, such as Ca deposition or osteoblastic differentiation, were less likely to be induced by calcifying medium in RAW 264.7 cells compared with mesothelial cells or fibroblasts (Supplementary Fig. S5).

## Discussion

**Figure 5:**
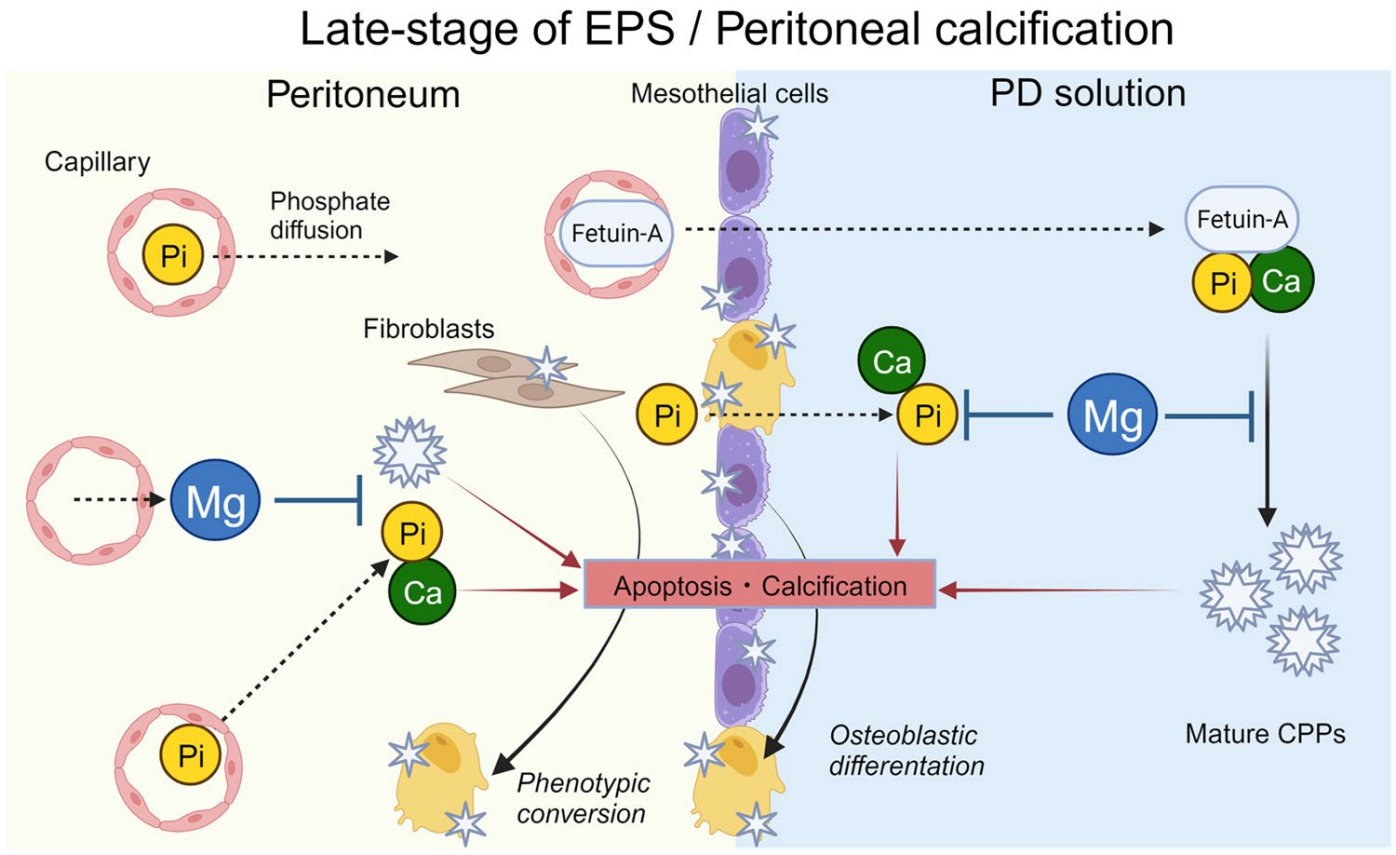
Schematic diagram of pathogenesis and preventive strategy of peritoneal calcification. Peritoneal calcification is regarded as a late-stage characteristic of EPS. Exposure of the peritoneum to high calcium and phosphate conditions results in peritoneal calcification through the conversion of peritoneum-constituting cells into osteoblast-like cells and increased cell death, which can be prevented by magnesium supplementation. *Ca* calcium, *CPP* calciprotein particle, *EPS* encapsulating peritoneal sclerosis, *Mg* magnesium, *PD* peritoneal dialysis, *Pi* phosphate.

The peritoneal membrane in patients undergoing PD is constantly exposed to a high-phosphate, high-Ca environment. VC is accelerated by high-phosphate and high-Ca conditions^4, 5, 7^ and prevented by Mg treatment in patients undergoing hemodialysis or PD. We therefore hypothesized that calcification of the peritoneal membrane resembled the pathogenesis of VC and that Mg supplementation might prevent peritoneal calcification. In the present study, the appearance of RUNX2-positive cells, indicative of osteoblastic differentiation, was confirmed in the visceral peritoneum obtained from patients with EPS. Intraperitoneal injection of LPS and phosphate- and Ca-rich solution in combination with CKD induction by feeding an adenine-containing diet calcified the parietal peritoneum in mice in vivo, while excised mouse peritoneum cultured in calcified medium became heavily calcified ex vivo. In addition, a high-Ca, high-phosphate medium increased calcification of the extracellular matrix, accompanied by enhanced osteochondrogenic transdifferentiation and cell death in cultured mesothelial cells and fibroblasts in vitro. Notably, the calcification of both cultured peritoneum-constituting cells and excised mouse peritoneum was markedly reduced by high-Mg conditions. A schema of the proposed pathophysiology of peritoneal calcification and the protective effects of Mg is shown in Fig. 5.

Fibrosis and angiogenesis are characteristic histological features of PD-related peritoneal damage. Most previous studies investigating the pathogenesis of EPS, as the most advanced form of peritoneal injury, have focused on angiogenesis^12–15^. The typical radiographic findings of EPS include peritoneal calcification, bowel thickening, tethering, and dilatation^3, 16^. Notably however, the pathological mechanism of peritoneal calcification has not been fully investigated. We hypothesized that phosphate and Ca overload may play a significant role in the pathogenesis of peritoneal calcification in the late stage of EPS. Although continuous ambulatory peritoneal dialysis is presumed to be better at controlling hyperphosphatemia than hemodialysis^17^, a previous survey showed that hyperphosphatemia also occurred frequently in patients undergoing PD, especially among anuric patients^18^. EPS occurs after withdrawal from PD in most (70–90% in some series) patients^19, 20^, with a lag from the cessation of PD until the development of EPS of up to 5 years^21^. Considering the large amount of phosphate removed by PD (approximately 2300 mg/week)^22^, the lifetime phosphate exposure of the peritoneum in PD patients is expected to reach 600 g (19,355 mmol). These findings support our investigations of the impact of pathological phosphate exposure on the peritoneum. In contrast, it is more difficult to calculate Ca exposure compared with phosphate exposure to the peritoneum in PD patients, because transperitoneal Ca transfer is determined by the patient’s serum Ca levels, the Ca concentration in the peritoneal dialysate, and transperitoneal convection volume^23^. Indeed, Ca can even be transferred from the peritoneal dialysate to the patient if the Ca concentration in the dialysate is high^24^. The impacts of Ca loading on peritoneal calcification may thus depend on patient-related factors.

We investigated if calcifying solution increased the peritoneal Ca content in vivo and found that, although calcifying solution alone did not affect peritoneal calcification, additional LPS administration did tend to increase the peritoneal Ca content. An LPS-induced peritonitis model is often used to mimic PD-related peritonitis^25, 26^. Intraperitoneal administration of LPS, an integral component of the outer membrane of Gram-negative bacteria, initiates rapid and coordinated recruitment and activation of leukocytes and macrophages, followed by the overproduction of proinflammatory mediators^27^. Results from in vitro studies and animal models of atherosclerosis suggested that inflammatory cytokines promoted VSMC differentiation and vascular intimal calcification^28–30^. In our in vitro study, LPS-activated macrophage supernatant induced mesothelial cell apoptosis, suggesting that the inflammatory response induced by peritonitis may accelerate peritoneal calcification via mesothelial cell apoptosis. However, the cultured macrophages did not calcify significantly in our in vitro study, suggesting that a direct phenotypic change of macrophages into osteoblast-like cells might not have contributed to the pathogenesis of peritoneal calcification.

Peritoneal mesothelial cells exposed to peritoneal dialysate exhibit diverse reactions. Prolonged exposure of mesothelial cells to hyperosmotic conditions, hyperglycemia, high concentrations of glucose-degradation products, low pH dialysate, and repeated peritonitis causes peritoneal injury with progressive mesothelial cell denudation, neo-angiogenesis, and fibrosis, loss of epithelial phenotype, and acquisition of fibroblast-like characteristics^31, 32^. However, few studies have determined if exposure to high-phosphate conditions induces a phenotypic change in mesothelial cells. The present study demonstrated that calcifying medium with a high phosphate and Ca concentration increased mRNA levels of osteoblastic differentiation markers, and confirmed that apoptosis, as another important mechanism of the calcification process, was also enhanced in mesothelial cells and fibroblasts, consistent with previous reports^33, 34^. Overall, these results indicate that a high-phosphate environment leads to calcification of the extracellular matrix in mesothelial cells and fibroblasts by accelerating at least two major steps of VC. A previous study proposed a “two-hit theory” to explain the development of EPS, in which the peritoneum deteriorates by exposure to PD solution (first hit) and is then exposed to additional deteriorating factors (second hit)^35^. The current LPS-activated macrophage supernatant experiment might be related to the typical second hit. Peritoneal thickness, as an indicator of peritoneal fibrosis correlated with angiogenesis^36^, was significantly increased in mice with peritoneal calcification. We also confirmed that a high-phosphate environment caused mesothelial cell death, reflecting mesothelial cell detachment from the peritoneum as a feature of EPS^35^. This result was consistent with a previous report showing phosphate-induced apoptosis of peritoneal mesothelial cells^34^. Moreover, we showed that calcifying medium tended to promote VEGF-A and TGFβ1 secretion associated with angiogenesis and fibrosis in mesothelial cells. These results indicate that a high flow of inorganic phosphate into the peritoneal cavity may serve as another first-hit factor of EPS.

CPPs have been reported to be directly involved in inflammation and uremic VC^37^. Fetuin-A modifies the pro-calcific potential of CPPs and prevents VC^38^. Intriguingly, a previous proteomics study of PD effluent revealed that fetuin-A levels were increased 1 year before EPS development, and could act as a marker for the early diagnosis of EPS^39^. Furthermore, another researcher isolated CPPs from PD effluent^8^. In line with these reports, although we did not examine CPPs directly in the in vivo study, the present study suggested that CPPs formed in high-Ca, high-phosphate conditions in the peritoneal cavity might have induced peritoneal calcification in our animal model. The calcifying medium accelerated the formation of mature CPPs, which was suppressed by medium containing 1.5 mM Mg in our in vitro study. Given that Mg inhibits CPP-driven hydroxyapatite formation and VC^40^, increasing the Mg ion concentration in the PD solution may inhibit the formation of secondary CPPs, thereby preventing peritoneal calcification. In the present study, serum Mg concentrations were similar between the two CKD groups, suggesting that local Mg supplementation can effectively inhibit peritoneal calcification without increasing serum Mg levels.

Increasing evidence has revealed a protective effect of Mg against ectopic calcification and inflammation. Lower serum Mg levels significantly predicted high mortality and VC progression in PD patients^41, 42^. A previous in vitro study showed that Mg strongly inhibited phosphate-induced osteochondrogenic differentiation and apoptosis of VSMCs and inhibited the production of profibrotic and proinflammatory cytokines^43^. Notably, phosphate and Mg had opposite effects on the mitochondrial permeability transition pore (mPTP), which regulates mitochondria-mediated cell death; phosphate promoted mPTP opening followed by activation of the mitochondrial apoptotic pathway, while Mg inhibited this pathway^44–46^. In the current study, Mg suppressed phosphate-induced apoptosis and osteogenic transdifferentiation in mesothelial cells and fibroblasts. Our data provide robust evidence supporting Mg as a promising therapeutic option for the prevention of EPS progression in patients receiving PD.

Notably, the Mg concentration in clinical peritoneal dialysate is relatively low (0.25–0.75 mEq/L) for the purpose of Mg elimination^42, 47, 48^. One cohort study showed mean serum Mg levels of 0.84 mEq/L in PD patients, and > 32% of this cohort had low serum Mg levels (defined as < 0.74 mEq/L). Although some reports mentioned the need for Mg supplementation in PD patients^42, 48^, parenteral Mg supplementation is often linked to gastrointestinal side effects, thereby lowering adherence. Given that intraperitoneal Mg supplementation has not been linked to gastrointestinal side effects, increasing the Mg concentration in the peritoneal dialysate may be a promising approach for preventing high-phosphate-and high-Ca induced peritoneal damage, ultimately suppressing EPS progression and improving mortality in PD patients.

CKD might have aggravated the peritoneal calcification induced by the calcifying solution in our mouse model of peritoneal calcification via several potential mechanisms. First, CKD might have accelerated peritoneal calcification by decreasing fetuin-A levels. In the present study, we employed a diet inducing CKD and malnutrition^49^. Malnutrition–inflammation complex syndrome, a typical pathophysiology of CKD, contributes to the development of VC and sarcopenia^50, 51^. Serum fetuin-A levels are decreased in patients with CKD and malnutrition^52^. Fetuin-A protects against VC by inhibiting the generation and maturation of CPPs^53^, and CKD can thus promote peritoneal calcification. Second, hyperphosphatemia induced by CKD might have increased phosphate loading and mature CPPs. Third, as shown in the pathogenesis of VC, CKD might have enhanced systemic and peritoneal inflammation, thereby promoting peritoneal calcification^54, 55^. More studies focusing on the impact of CKD on peritoneal calcification are needed to further our understanding of the pathophysiology of EPS in PD patients.

Peritoneal calcification may indicate an effective strategy for preventing the progression of VC. In the present study, Ca overload augmented high-phosphate-induced calcification of the extracellular matrix in cultured MeT5A cells, suggesting that the simultaneous unloading of phosphate and Ca could protect against peritoneal calcification. Dietary phosphate restriction, phosphate binder use, and the use of calcimimetics to prevent secondary hyperparathyroidism may prevent hyperphosphatemia and decrease transperitoneal phosphate exposure, ultimately slowing the progress of peritoneal calcification^56^. Regarding Ca unloading, we should reduce the Ca concentration of the dialysate and minimize the use of vitamin D receptor activators and Ca-based phosphate binders^23, 57, 58^.

In conclusion, the current multifaceted in vivo, ex vivo, and in vitro approach, including human data, suggests that exposure of the peritoneum to high Ca and phosphate levels results in peritoneal calcification, through the conversion of peritoneum-constituting cells into osteoblast-like cells and increased cell death, which can be prevented by Mg supplementation. Further experimental studies are needed to determine if increasing the Mg concentration in the peritoneal dialysate represents a promising therapeutic approach to prevent the progression of peritoneal cell death, fibrosis, and calcification in patients undergoing PD.

## Materials and methods

The detailed methods are available as Supplementary Material S1 on the Scientific Reports’ website.

### Peritoneum samples from patients with encapsulating peritoneal sclerosis

Visceral peritoneal tissues were obtained from two patients diagnosed with EPS and one control patient undergoing hemodialysis at Karatsu Red Cross Hospital. We performed immunohistochemical staining of the osteoblastic differentiation marker RUNX2 and MSLN, which is expressed in mesothelial cells of the visceral peritoneum. The study protocol was performed according to the Ethics of Clinical Research (Declaration of Helsinki) and approved by the Local Ethics Committee of Kyushu University Hospital (No. 2022-148). Informed consent was obtained from all subjects and/or their legal guardian(s).

### Animal experiment

All animal experiments were approved by the Ethics Committee on Animal Experimentation, Kyushu University Graduate School of Medical Sciences (approval number A20-245-1) and reported in accordance with ARRIVE (Animal Research: Reporting of In Vivo Experiments) guidelines (https://arriveguidelines.org). All experiments were performed in accordance with relevant guidelines and regulations. Male C57BL/6Jcl mice aged 9 weeks were purchased from CLEA Japan, Inc. (Tokyo, Japan). Control mice received 10 mL/kg/day saline, and peritoneal calcification model mice received calcifying solution (2.5 mM Ca, 3.5 mM phosphate) with or without 1.5 mM Mg (Nacalai Tesque Inc., Kyoto, Japan) injected intra-abdominally at a total volume of 10 mL/kg body weight for 21 consecutive days, plus intra-abdominal injection of LPS10 mg/kg (Sigma-Aldrich Japan, Tokyo, Japan) on day 1^25, 26^. Mice were fed a customized CKD diet containing 0.2% adenine, 1.0% Ca, and 1.2% phosphate^49^ (KBT Oriental Co., Ltd., Saga, Japan). The experimental protocols are shown in Supplementary Fig. S6. For ex vivo experiments, the parietal peritoneum was excised from 12-week-old untreated male mice and incubated in calcifying medium with 0.8 mM or 1.5 mM Mg for 72 h (Supplementary Fig. S6). Mice were euthanized on day 28 by intraabdominal injection of 0.3 mg/kg medetomidine hydrochloride (Orion Corporation, Espoo, Finland), 4 mg/kg midazolam (Sandoz, Tokyo, Japan), and 5 mg/kg butorphanol tartrate (Meiji Seika, Tokyo, Japan). Twenty milliliters of ice-cold phosphate-buffered saline (PBS, pH 7.4) were slowly perfused to harvest the peritoneum. The detailed methods are described in the Supplementary Materials and Methods S1. All the experiments were carried out following the relevant guidelines and regulations.

### Cell lines and culture

We used MeT5A human mesothelial cells (American Type Culture Collection, Manassas, VA, USA) and MEFs (Riken Cell Bank, Tsukuba, Japan). Cells were incubated overnight in serum-free medium followed by incubation in calcifying medium (2.5 mM Ca, 3.5 mM phosphate, 0.8 mM Mg) or calcifying medium plus high Mg (1.5 mM) for 72 h (Supplementary Fig. S6). The concentrations of Ca, phosphate, and Mg used in the in vitro, ex vivo, and in vivo experiments were determined based on previous reports and clinical settings^43, 49^. The concentrations of Ca and Mg used in clinical practice range from 2.5–3.5 mM and 0.5–1.5 mM, respectively. The Ca, phosphate, and Mg concentrations used in the present study were therefore considered reasonable. RAW 246.7 mouse macrophages (American Type Culture Collection) were also cultured to investigate the effect of inflammatory cytokines secreted by macrophages on mesothelial cells. RAW264.7 cells were incubated in 0.1 μg/mL LPS for 24 h. The supernatant was recovered and centrifuged at 3000×*g* for 10 min and the collected supernatant was used to stimulate MeT5A cells (Supplementary Fig. S6).

### Immunofluorescence, real-time polymerase chain reaction, western blotting, and enzyme-linked immunosorbent assay

Immunohistochemical analysis of tissues and cultured cells, quantitative reverse transcriptase polymerase chain reaction, western blotting, and enzyme-linked immunosorbent assay were conducted by standard methods (Supplementary Materials and Methods S1).

### Statistical analysis

Data are expressed as mean ± standard deviation. Parametric variables were compared between groups using the Mann–Whitney U-test, and differences among groups were compared by one-way analysis of variance, followed by Tukey’s post hoc test. All statistical analyses were performed using R version 4.0.2 (http://cran.rproject.org). A two-tailed value of *P* < 0.05 was considered statistically significant.

### Supplementary Information

Supplementary Information.

## Data Availability

The datasets generated during and/or analyzed during the current study are available from the corresponding author on reasonable request.
