# Supplementary material for: Magnesium inhibits peritoneal calcification as a late-stage characteristic of encapsulating peritoneal sclerosis
Source: Sci Rep. 2023 Sep 28;13:16340. doi: 10.1038/s41598-023-43657-y (PMC10539370; doi:10.1038/s41598-023-43657-y)
Supplement: Supplementary file 1 — Supplementary Information. [file 41598_2023_43657_MOESM1_ESM.pdf]

Supplementary Information Online

Magnesium inhibits peritoneal calcification as a late-stage characteristic of encapsulating peritoneal sclerosis

Seishi Aihara[1], Shunsuke Yamada[1], Shumei Matsueda[1], Akinori Nagashima[2], Kumiko Torisu[1], Takanari Kitazono[1], Toshiaki Nakano[1]*

[1]Department of Medicine and Clinical Science, Graduate School of Medical Sciences, Kyushu University, Fukuoka, Japan

[2]Department of Nephrology, Karatsu Red Cross Hospital, Saga, Japan

Corresponding author

Toshiaki Nakano, M.D., Ph.D.

Department of Medicine and Clinical Science, Graduate School of Medical Sciences, Kyushu University, 3-1-1 Maidashi, Higashi-Ku, Fukuoka 8128582, Japan.

Tel: +81-92-642-5843; Fax: +81-92-642-5846

E-mail: nakano.toshiaki.455@m.kyushu-u.ac.jp

Supplementary Materials and Methods

Peritoneum Samples from Patients with EPS

Peritoneum samples were obtained from two patients diagnosed with EPS and from control patients undergoing hemodialysis at Karatsu Red Cross Hospital. One EPS patient was a 65-year-old male who had received PD for 7 years and the other was a 60-year-old female who had received PD for 11 years. Both of these patients died of EPS.

Sample Collection and Histological Examination

Parietal peritoneum samples were snap-frozen in liquid nitrogen and stored at −80ºC for *in vivo* experiments. Excised parietal peritoneum samples were fixed in neutral-buffered 10% formalin (Wako, Osaka, Japan), embedded in paraffin, and used for further histological studies. Four-micrometer paraffin sections were stained with Alizarin Red (Fujifilm Corporation, Tokyo, Japan) to detect peritoneal calcification. Serum Mg concentrations in the *in vivo* study were measured using the Magnesium B-test (Fujifilm Corporation), according to the manufacturer's instruction. Peritoneal thickness was determined using a micrometer and analyzed using ImageJ software. The peritoneal thickness was calculated by averaging the peritoneal thickness measured at nine randomly selected points, as reported previously [S1].

Cell Lines and Culture

Cells were cultured in Dulbecco’s Modified Eagle Medium (DMEM, Nacalai Tesque) containing 10% fetal bovine serum (Biowest, Nuaillé, France), 100 U/mL penicillin, and 100 mg/mL streptomycin (Life Technologies, Carlsbad, CA, USA), in a humidified atmosphere

with 5% CO_2 at 37°C. The cells were seeded onto 12-well culture plates at a density of 5.0×10^4 cells/mL for MeT5A cells and 1.0×10^4 cells/mL for MEFs.

Analyses of Extracellular Matrix Calcification (*in Vivo, ex Vivo, and in Vitro*)

Peritoneum samples from *in vivo* and *ex vivo* experiments were homogenized twice with 6.0 mol/L hydrochloride (Wako) for 3 min each at 30 Hz using a Tissue-Lyser (Qiagen K.K., Tokyo, Japan). MeT5A cells and MEFs were decalcified in 0.6 mol/L hydrochloride at 4°C overnight, followed by protein extraction with 0.2 mol/L sodium hydroxide (Wako) for 1 h. The supernatants of the hydrochloride- and sodium hydroxide-treated solution were analyzed by Calcium E-test (Fujifilm Corporation, Tokyo, Japan) and BCA protein assay (Thermo Scientific, Waltham, MA, USA), according to the manufacturer's instruction. Calcium deposition in the extracellular matrix of the peritoneum and cells was determined by calcium content normalized to tissue dry weight and protein content (mg/g protein), respectively. Serum levels of urea nitrogen, creatinine, albumin, calcium, and phosphate were measured using an automated analyzer (DRI-CHEM; Fujifilm Corporation). Serum levels of cystatin C were measured using an enzyme-linked immunosorbent assay (ELISA) kit (Abcam, Cambridge, UK), according to the manufacturer's instruction.

Immunofluorescence

Sections from paraffin blocks of human peritoneum and frozen blocks from *in vivo* experiments were stained. Human visceral peritoneum specimens embedded in paraffin were immunostained after antigen retrieval, using antigen activation solution (415211; Nichirei Biosciences Inc., Tokyo, Japan) and heat-induced epitope. The following primary antibodies

were used: rabbit RUNX2 polyclonal antibody (sc-10758, 1:200, Santa Cruz Biotechnology, Dallas, TX, USA), mouse MSLN antibody (66404, 1:200, Proteintech Group, Inc.), mouse α-SMA antibody (ab7817, 1:200, Abcam), and rabbit F4/80 antibody (30325S, 1:200, Cell Signaling). Secondary antibodies were as follows: Alexa 488-conjugated goat anti-rabbit IgG (A11008, 1:500, Life Technologies) and Alexa 568-conjugated goat anti-mouse IgG (A11031, 1:500, Life Technologies). Tissues were counterstained with 4′,6-diamino-2-phenylindole (DAPI) and images were obtained by fluorescence microscopy (BX53; Olympus). Cells were mounted and counterstained with VECTASHIELD Mounting Medium containing DAPI (Vector Laboratories, Burlingame, CA, USA). Immunofluorescence signals were visualized using a Zeiss LSM 700 confocal microscope (Carl Zeiss, Germany).

Real-time Polymerase Chain Reaction (RT-PCR)

Total RNA was extracted from cells using a MAXWELL®16 LEV simply RNA tissue kit (Promega, Madison, WI, USA) and MAXWELL®16 instrument (Promega) according to the manufacturer's instructions. Complementary DNA was synthesized from 1 µg of total RNA using a PrimeScript RT Reagent Kit (Takara Bio Inc.). RT-PCR was performed using SYBR Premix Ex Taq™ (Takara Bio Inc.) and a 7500 Real-Time PCR System (Applied Biosystems, Foster City, CA, USA). Relative expression levels of human RUNX2 (forward 5′-TCCACACCATTAGGGACCATC-3′, reverse 5′-TGCTAATGCTTCGTGTTTCCA-3′), human BMP2 (forward 5′-CTGGCTGATCATCTGAACTCCACT-3′, reverse 5′-CTCGTCAAGGTACAGCATCGAGAT-3′), human OPN (forward 5′-AGGAAACCAGCCAAGGACTAAC-3′, reverse 5′-GGCAATGCCAAACAGGCAAA-3′), mouse OPN (forward 5′-CTGGCAGCTCAGAGGAGAAG-3′, reverse 5′-

GGACATCGACTGTAGGGACG-3′), and mouse α-SMA (forward 5′-TTCGTGTGGCCCCTGAAGAGCAT-3′, reverse 5′-CCAGTTGTACGTCCAGAGGCA-3′) in cells were determined by the ΔΔCT method using human (forward 5′-AAACGGCTACCACATCCAAG-3′ and reverse 5′-CCTCCAATGGATCCTCGTTA-3′) or mouse (forward 5′-AAGTTTCAGCACATCCTGCGAGTA-3′ and reverse 5′-TTGGTGAGGTCAATGTCTGCTTTC-3′) ribosomal protein 18 s as an internal reference.

Western Blotting

The cell lysate was extracted using lysis buffer (mammalian protein extraction reagent, M-PER™; Thermo Scientific, with a protease inhibitor cocktail; Nacalai Tesque), centrifuged at 10,000 ×*g* for 10 min at 4°C, and the collected supernatant was analyzed. Protein samples (10 µg) were separated by sodium dodecyl sulfate-polyacrylamide gel electrophoresis on 5%–20% polyacrylamide gradient gels (PAGEL; Atto, Tokyo, Japan) and blotted onto a polyvinylidene difluoride membrane using a Trans-Blot Turbo System (BioRad, AbD Serotec, Oxford, UK). Primary and secondary antibodies were diluted in antibody solution (signal enhancer HIKARI, Nacalai Tesque). After preincubation in blocking solution (Blocking One, Nacalai Tesque) for 30 min, the membranes were incubated overnight at 4°C with primary antibodies to rabbit cleaved caspase-3 (1:1000, Cell Signaling Technology) and rabbit anti-αβ tubulin (1:5000, Cell Signaling). After washing three times in Tris-buffered saline with Tween 20 (TBS-T), the membranes were incubated with horseradish peroxidase-conjugated anti-rabbit IgG secondary antibody (NA934, GE Healthcare, Bucks, UK) for 1 h. The bands were detected using an enhanced chemiluminescent kit (11644-40, Chemi-Lumi One Ultra; Nacalai Tesque) and captured using a chemiluminescence imaging system (AE-

9300 Ez capture MG; Atto). The density of each band was analyzed by ImageJ software (National Institutes of Health).

ELISA

The cell supernatant was recovered and centrifuged at 3,000 ×*g* for 10 min at 4ºC and the collected supernatant was analyzed. Vascular endothelial growth factor-A (VEGF-A) and transforming growth factor-β1 (TGFβ1) concentrations in the supernatant were measured using a human VEGF-A ELISA kit (ab119566, Abcam) and human TGFβ1 Quantikine ELISA kit (DB100B; R&D Systems), respectively.

Transmission electron microscopy

MET5A cells were incubated in DMEM supplemented with calcium, phosphate, and/or magnesium. The solution was applied to Formvar (polyvinyl formal)-coated 150 mesh (Nisshin-EM Inc., Japan), and the mesh was dried at room temperature without staining. The formation of CCPs in the culture medium was confirmed by visualization with a TH-7700 electron microscope (Hitachi, Japan) at 80 kV.

Statistical Analysis

Parametric variables with a normal distribution were expressed as mean ± standard deviation. Differences between two groups were compared using the Mann–Whitney U-test and differences among groups were compared by one-way analysis of variance (ANOVA), followed by Tukey’s *post hoc* test. All statistical analyses were performed using EZR software (Saitama Medical Center, Jichi Medical University, Saitama, Japan), which is a

graphical user interface for R (The R Foundation for Statistical Computing, Vienna, Austria), and a modified version of R commander designed to add statistical functions frequently used in biostatistics [S2]. A two-tailed value of $P<0.05$ was considered statistically significant. Graphs were created using GraphPad Prism version 6.0 for Windows (GraphPad Software).

References

S1. Honda K, Hamada C, Nakayama M, et al. Impact of Uremia, Diabetes, and Peritoneal Dialysis Itself on the Pathogenesis of Peritoneal Sclerosis: A Quantitative Study of Peritoneal Membrane Morphology. *Clin J Am Soc Nephrol* **3**, 720-728 (2008).

S2. Kanda Y. Investigation of the freely-available easy-to-use software “EZR” (Easy R) for medical statistics. *Bone Marrow Transplant* **48**, 452-458 (2013).

Supplementary Figures and Figure Legends

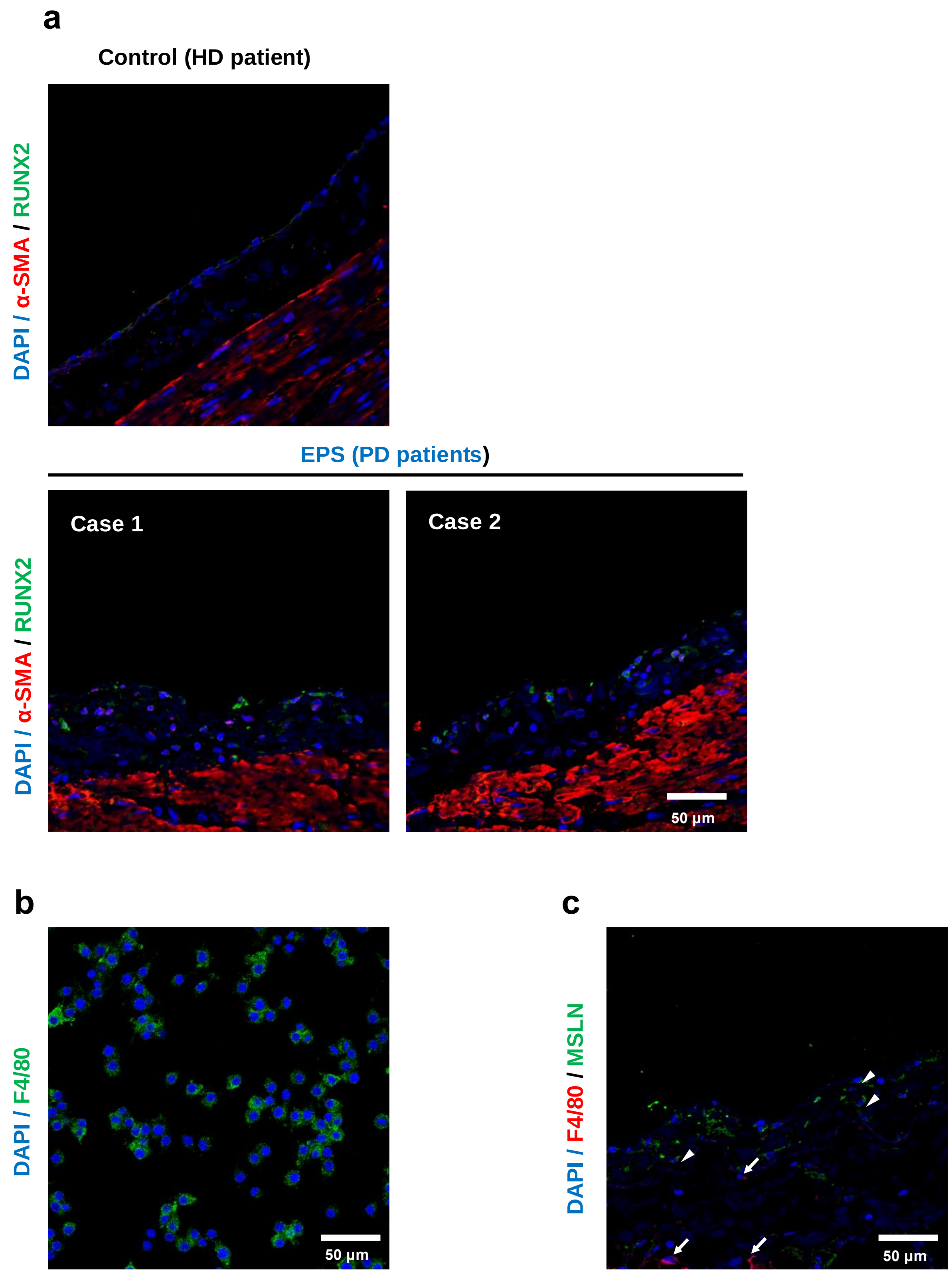

Supplementary Figure 1. Microphotographs of dual immunohistochemical staining of α-SMA and RUNX2 in peritoneum samples from two PD patients with EPS and one HD patient.

(a) Representative microphotographs of dual immunohistochemical staining of α-SMA and RUNX2 in peritoneum samples from two PD patients with EPS and one HD patient. α-SMA stained red, RUNX2 stained green, and DAPI colored blue. Scale bars, 50 µm. (b) Immunofluorescence images of F4/80 in RAW 264.7 cells. Green, anti-F4/80 antibody; blue, DAPI. Scale bars, 50 µm. (c) Representative microphotographs of dual immunohistochemical staining of F4/80 and MSLN in peritoneum samples from an EPS patient. Red, anti-F4/80 antibody; green, anti-MSLN antibody; blue, DAPI. White arrows indicate F4/80-positive macrophages; white arrowheads indicate mesothelial cells. Scale bars, 50 µm. Abbreviations: DAPI, 4',6-diamidino-2-phenylindole; EPS, encapsulating peritoneal sclerosis; HD, hemodialysis; α-SMA, alpha-smooth muscle actin; MSLN, mesothelin; PD, peritoneal dialysis; RUNX2, runt-related transcription factor 2.

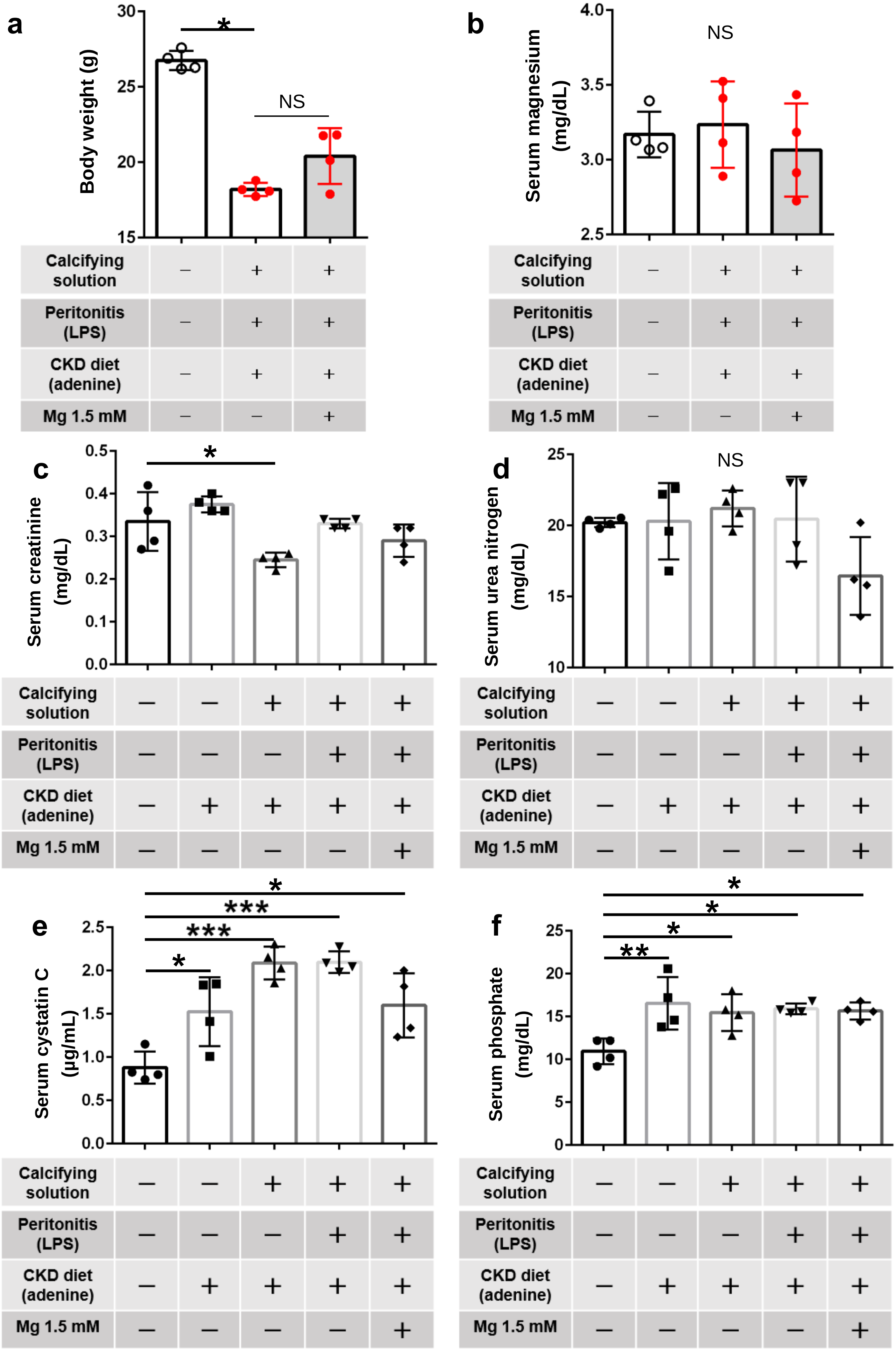
a
Body weight (g)
*
NS
b
Serum magnesium (mg/dL)
NS
c
Serum creatinine (mg/dL)
*
d
Serum urea nitrogen (mg/dL)
NS
e
Serum cystatin C (μg/mL)
*
***
***
*
f
Serum phosphate (mg/dL)
*
*
*
**
Calcifying solution
Peritonitis (LPS)
CKD diet (adenine)
Mg 1.5 mM

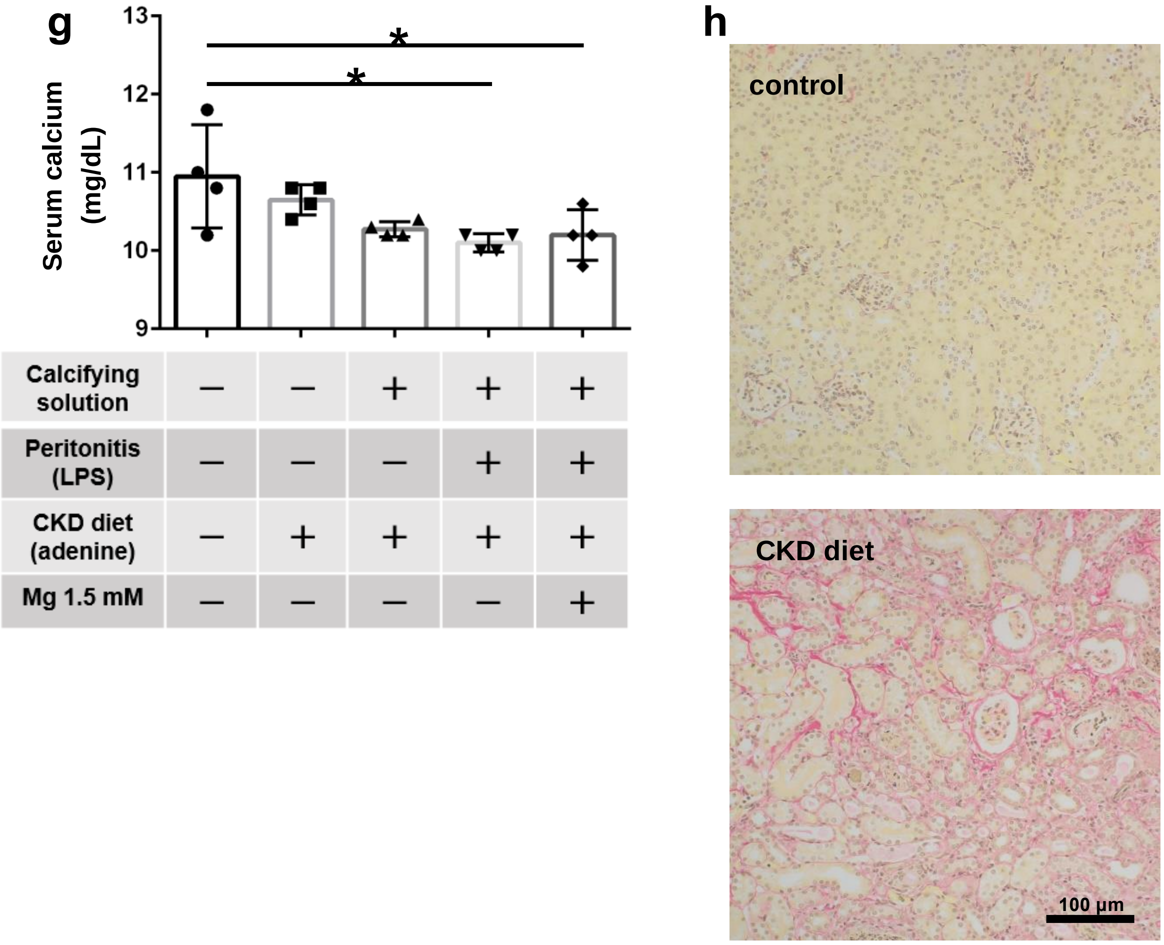

Supplementary Figure 2. Supplementary data for the *in vivo* study

(a) Body weight and (b) serum Mg concentrations in the three groups. Serum levels of (c) creatinine, (d) urea nitrogen, (e) cystatin C, (f) phosphate, and (g) calcium in the adenine-diet groups. *P<0.05, **P<0.01, ***P<0.001 (h) Representative images of Sirius Red staining in the kidney in mice administered an adenine diet. Scale bars, 100 μm. Abbreviations: CKD, chronic kidney disease; LPS, lipopolysaccharide; Mg, magnesium; NS, not significant.

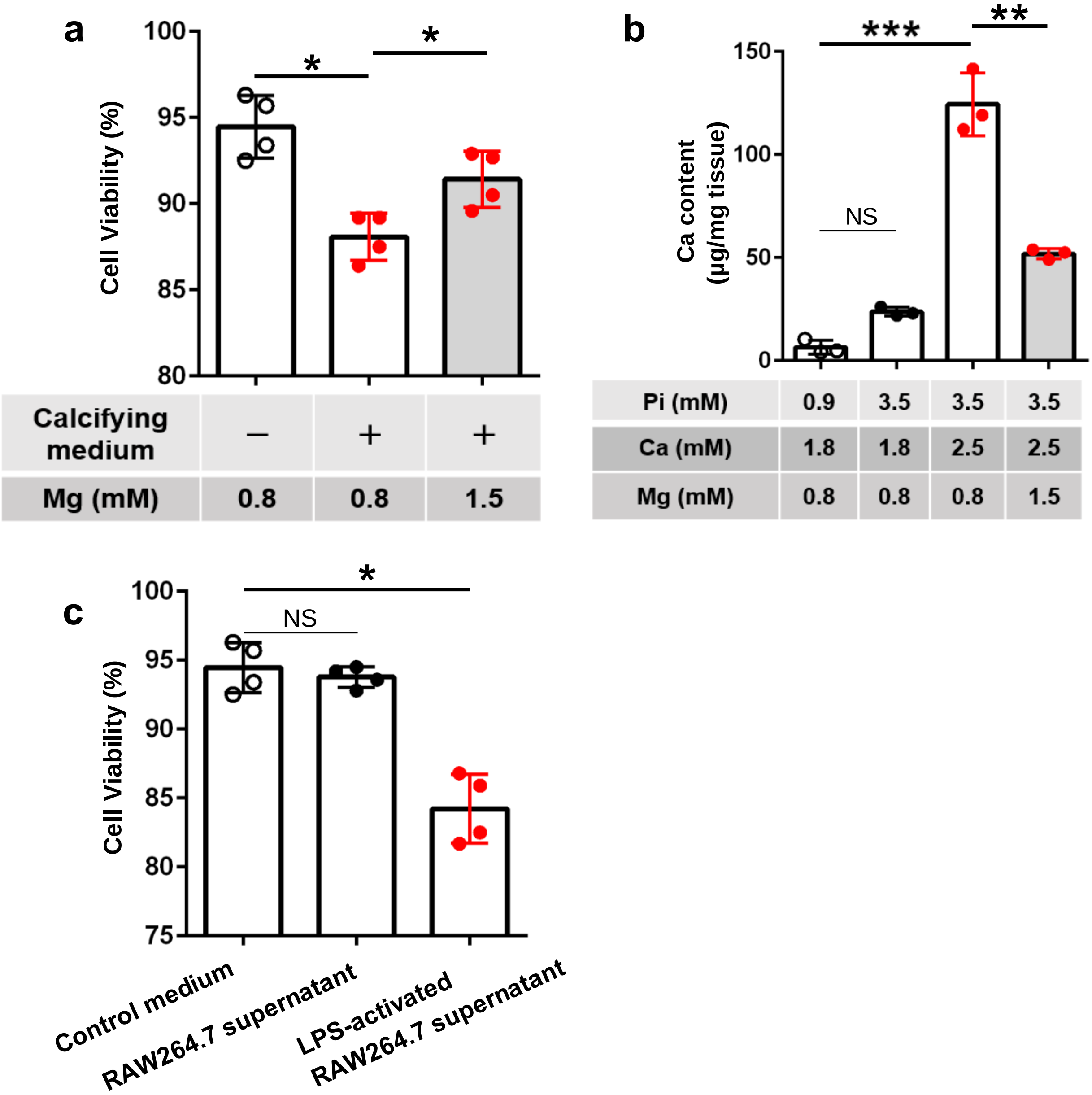

Supplementary Figure 3. Supplementary data for the *in vitro* study

(a) Cell viability determined by trypan blue staining in MeT5A cells incubated with calcifying medium (n=4 per group). (b) Quantitative analysis of calcification in MeT5A cells (n=3 per group). (c) Cell viability determined by trypan blue staining in MeT5A cells incubated with supernatant from LPS-activated RAW 264.7 cells (n=4 per group). Abbreviations: Ca, calcium; LPS, lipopolysaccharide; Mg, magnesium; Pi, phosphate; RAW 264.7, mouse macrophage cell line. *P<0.05, **P<0.01, ***P<0.001, NS, not significant.

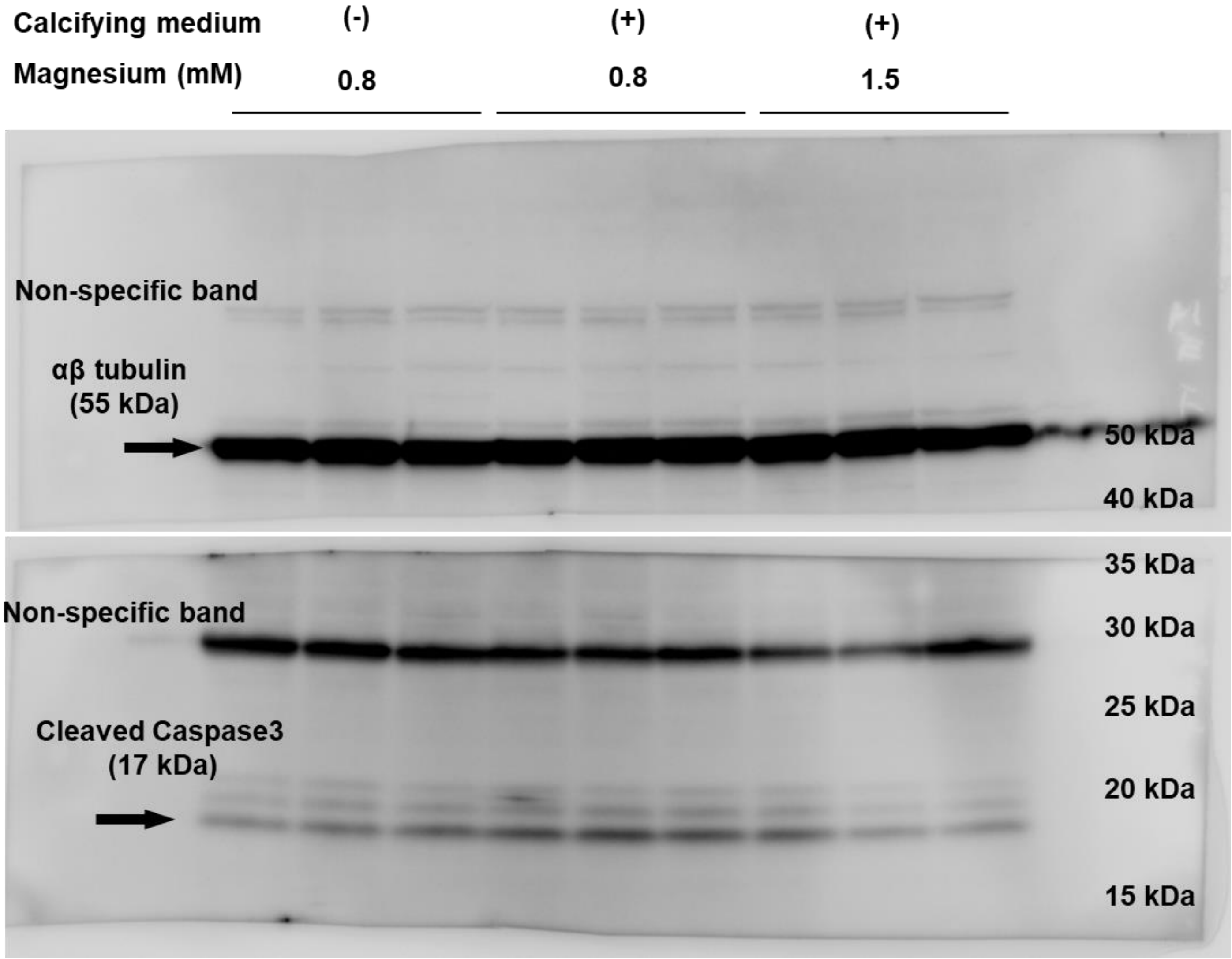

Supplementary Figure 4. Uncropped western blot image

Uncropped images of Figure 4e. Arrows show αβ tubulin (upper panel) and cleaved caspase 3 (lower panel) bands, respectively.

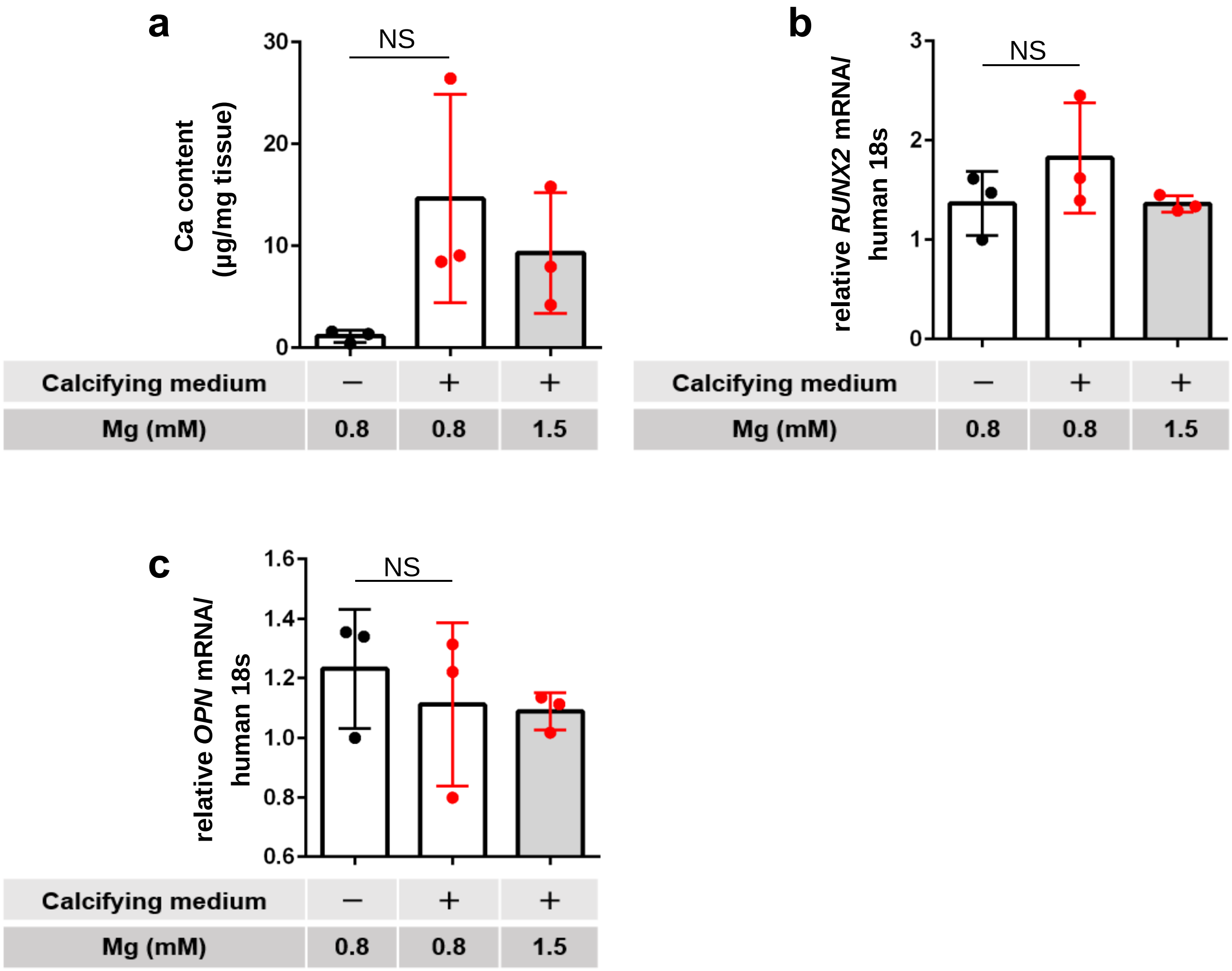

Supplementary Figure 5. Impact of calcifying medium on calcification of cultured macrophages

(a) Quantitative analysis of calcification in RAW 264.7 cells (n=3 per group). Relative mRNA expression levels of (b) RUNX2 and (c) OPN determined by real-time polymerase chain reaction in RAW 264.7 cells (n=3 for each). Abbreviations: Mg, magnesium; RAW 264.7, mouse macrophage cell line. NS, not significant; RUNX2, runt-related transcription factor 2; OPN, osteopontin.

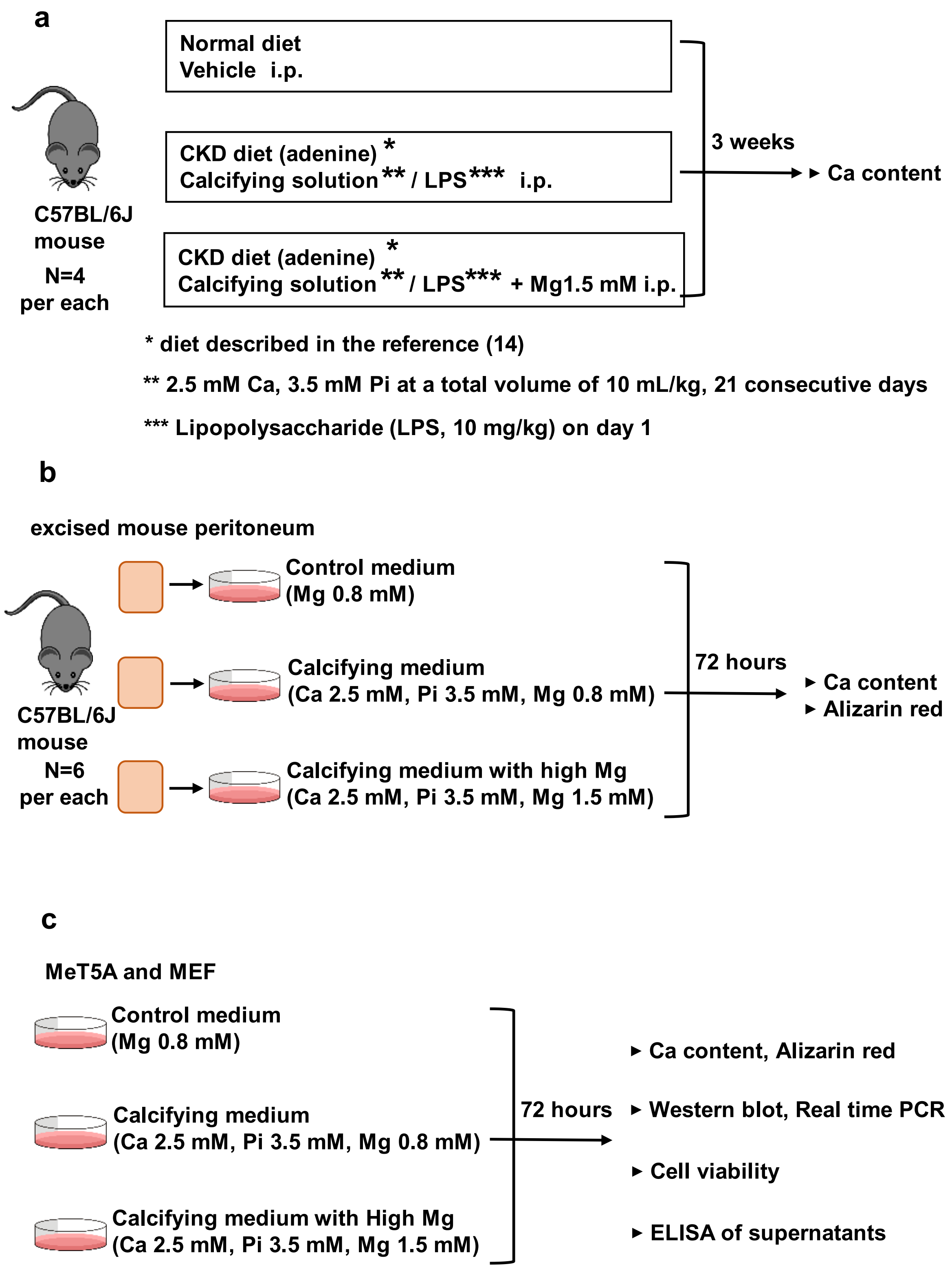
a
Normal diet
Vehicle i.p.
CKD diet (adenine) *
Calcifying solution ** / LPS *** i.p.
CKD diet (adenine) *
Calcifying solution ** / LPS *** + Mg1.5 mM i.p.
C57BL/6J mouse
N=4 per each
3 weeks
Ca content
* diet described in the reference (14)
** 2.5 mM Ca, 3.5 mM Pi at a total volume of 10 mL/kg, 21 consecutive days
*** Lipopolysaccharide (LPS, 10 mg/kg) on day 1
b
excised mouse peritoneum
Control medium (Mg 0.8 mM)
Calcifying medium (Ca 2.5 mM, Pi 3.5 mM, Mg 0.8 mM)
Calcifying medium with high Mg (Ca 2.5 mM, Pi 3.5 mM, Mg 1.5 mM)
C57BL/6J mouse
N=6 per each
72 hours
Ca content
Alizarin red
c
MeT5A and MEF
Control medium (Mg 0.8 mM)
Calcifying medium (Ca 2.5 mM, Pi 3.5 mM, Mg 0.8 mM)
Calcifying medium with High Mg (Ca 2.5 mM, Pi 3.5 mM, Mg 1.5 mM)
72 hours
Ca content, Alizarin red
Western blot, Real time PCR
Cell viability
ELISA of supernatants

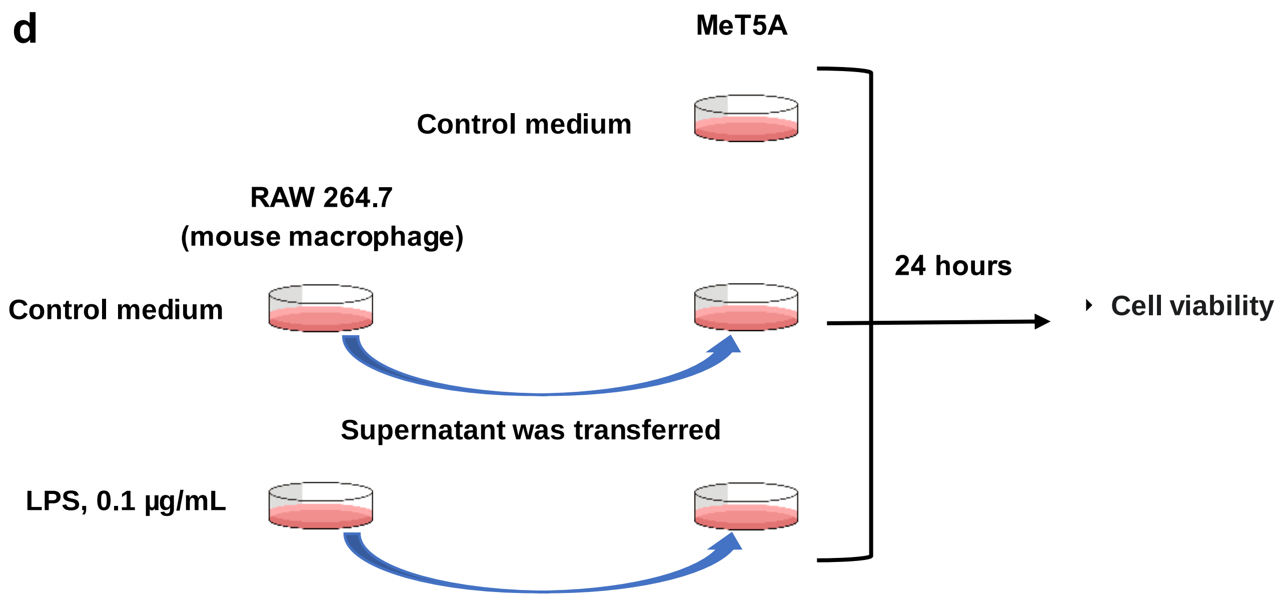

Supplementary Figure 6. Experimental protocol

(a) *In vivo* study using C57BL/6 mice (n=4 per group). (b) *Ex vivo* study using excised peritoneum from C57BL/6 mice (n=6 per group). (c) *In vitro* study using MeT5A cells and MEFs (n=3 per group). (d) *In vitro* study using supernatant from LPS-activated RAW 264.7 cells (n=3 per each group). Abbreviations: Ca, calcium; ELISA, enzyme-linked immunosorbent assay; i.p.; intraperitoneal administration; LPS, lipopolysaccharide; MEFs, cultured fibroblasts; MeT5As, cultured mesothelial cells; Mg, magnesium; RAW 264.7, mouse macrophage cell line; Pi, phosphate.
